# Effect of Resveratrol Supplementation on the SNARE Proteins Expression in Adipose Tissue of Stroptozotocin-Nicotinamide Induced Type 2 Diabetic Rats

**Published:** 2015-05

**Authors:** Azam Rezaei Farimani, Massoud Saidijam, Mohammad Taghi Goodarzi, Reza Yadegar Azari, Soheila Asadi, Sadegh Zarei, Nooshin Shabab

**Affiliations:** 1 Department of Biochemistry, School of Medicine, Hamadan University of Medical Sciences, Hamadan, Iran;; 2 Research Center for Molecular Medicine, Hamadan University of Medical Sciences, Hamadan Iran;; 3 Department of Genetic and Molecular Medicine, School of Medicine, Hamadan University of Medical Sciences, Hamadan, Iran

**Keywords:** GLUT4, SNAP23 Protein, Syntaxin-4 Protein, Vesicle-associated membrane Protein 2, Resveratrol

## Abstract

**Background:**

Glucose uptake by muscles and fat cells is carried out by the GLUT4 system. Isoforms of the SNAP23, syntaxin-4 and VAMP-2 play an important role in regulating GLUT-4 trafficking and fusion in adipocytes. The changes of SNARE proteins levels and thus impaired GLUT-4 displacement can be one of the etiological causes of type 2 diabetes.

Due to changes in the expression of these proteins in diabetes, the aim of this study was to investigate the effect of the natural compound resveratrol with anti-diabetic properties on impaired expression of SNARE proteins in type 2 diabetes.

**Methods:**

Forty male Wistar rats were used in this study. Type 2 diabetes was induced by administering a single dose of streptozotocin and nicotinamide. The expression of SNAP-23, syntaxin-4 and VAMP-2 proteins were assessed using real-time qRT-PCR. Also, some biochemical parameters were examined, including fasting blood glucose, insulin levels and insulin resistance.

**Results:**

The results of this study showed that, resveratrol supplementation increased blood insulin level, reduced the fasting blood glucose, and improved the insulin resistance. In addition, resveratrol supplementation increased the expression of SNAP-23, syntaxin-4 and VAMP-2 proteins that involved in GLUT-4 transport in adipose tissue of diabetic rats.

**Conclusion:**

Final results showed that SNARE proteins expression is significantly reduced in diabetic rats and treatment with resveratrol supplementation is associated with the increased expression of these proteins.

## Introduction

Insulin resistance and defective insulin secretion are the two leading pathological causes in type 2 diabetes. Insulin resistance is characterized by the inability of glucose uptake into muscle cells and adipocytes.^1^ Glucose uptake by muscle and fat cells is carried out by the GLUT4 system that is sequestered in an intracellular compartment. After stimulation by insulin, GLUT4 is quickly located on the cell surface and increase glucose transport into cells and thus maintain glucose homeostasis.^2^ Among the wide range of studies performed on proteins, the soluble N-ethylmaleimide-sensitive factor activating protein receptors, or SNAREs, play important roles in membrane trafficking, docking, and fusion.^3^ SNARE proteins are a ternary complex consisting of the v-SNARE protein vesicle-associated membrane protein (VAMP) that interacts with the t-SNARE proteins syntaxin and synaptosomal-associated protein of 25 kDa (SNAP-25).^4^^,^^5^ Studies have demonstrated isoforms of the SNAP-23, syntaxin-4 and VAMP-2 play important roles in regulating GLUT-4 traffic and vesicle fusion in adipocytes.^6^^,^^7^ Therefore, dysfunction of the SNARE complex inhibits translocation of GLUT4 to the cell surface and subsequently leads to increased blood glucose levels and insulin resistance. According to these findings, the changes of SNARE proteins levels and thus impaired GLUT-4 displacement can be one of the etiological causes of type 2 diabetes.^8^^,^^9^ Some studies have indicated that, agents that increase insulin sensitivity are able to modulate the levels of these modified proteins.^9^ In recent years, numerous studies have been conducted on natural factors affecting diabetes including resveratrol. Resveratrol (3, 5, 4′-trihydroxystilbene, RSV) is a polyphenol found in high levels in grapes and red wine.^10^ Nowadays, this is commercially available in the form of dietary supplementation tablets. Some studies on diabetic animal and human models have shown that this plant-derived compound affects insulin secretion and function, leading to reduce blood glucose. The studies have indicated different mechanisms of resveratrol function on glucose uptake, including enhanced expression and translocation of GLUT4 to the plasma membrane.^11^

Since it is recognized that impaired expression of SNARE proteins could be one of the causes of insulin resistance and consequently hyperglycemia in type 2 diabetes, the purpose of this study was to assess the effect of resveratrol supplementation on the expression of these proteins and to show a possible molecular mechanism of resveratrol action. 

Additionally, we examined the effect of resveratrol on insulin resistance index, serum glucose and serum insulin levels. 

## Materials and Methods

*Materials*

Resveratrol supplementation was from Amazon (USA), streptozotocin (STZ) and nicotinamide (NA) were purchased from Sigma Aldrich (Germany) and Rat Insulin (INS) ELISA kit was from Bio-equip (China).

*Animals*

Forty male Wistar rats (200-250 g) were used in this study, purchased from the animal house of Razi Institute, Iran. Handling and experiments on animals were according to the protocols approved by the Ethics Committee of Hamadan University of Medical Sciences. Rats were fed with fresh water and standard chow during the maintenance period and environmental conditions were maintained as standard condition with 12 hour light/dark cycles. Animals were divided into five groups of 8 rats in each group. Two groups of normal and diabetic rats were considered as controls and three groups of diabetic rats treated with different doses of resveratrol supplementation (1, 5, 10 mg/kg body weight/day) as cases.

*Induction of Type2 Diabetes*

To induce diabetes, rats were fasted overnight, and then 60 mg/kg streptozotocin (0.1 M in sodium citrate; pH, 4.5) was administered intraperitoneally. After 15 minutes, 120 mg/kg nicotinamide dissolved in normal physiological saline was injected intraperitoneally. Intraperitoneal injections (STZ+NA) were performed at a single dose.^12^ In order to confirm diabetes in rats, 72 h after injection, fasting blood glucose (tail vein) levels was assessed using a glucometer. Rats with fasting blood glucose levels higher than 150 mg/dL were considered as diabetic.^13^

*Treatment and Sample Collection*

Resveratrol supplementation treatment was initiated seven days after diabetes induction. The various doses of resveratrol (1, 5, 10 mg/kg) were dissolved in distilled water and injection was performed orally using a gavage syringe.^14^ The case groups were treated with resveratrol supplementation for a month. After completion of the treatment, each rat was anesthetized by Ketamine: Xylazine (100 mg/kg: 5-10 mg/kg IP),^15^ then visceral adipose tissue samples were separated from all animals. Cryotubes containing samples were immediately frozen in liquid nitrogen and were transferred at -80°C until analysis. For the analysis of biochemical parameters, serum prepared from blood samples taken from the cardiac puncture and stored at -20°C.

*Total RNA Extraction*

In order to extract RNA, frozen tissues (~300 mg) were homogenized in liquid nitrogen using pestle. RNA Extraction was performed manually using Trizol (Invitrogen).^16^ Completely crushed tissues were transferred to a tube containing 1ml Trizol and incubated for 10 minutes at room temperature. In order to separate the insoluble debris, centrifuge at 12,000×g for 10 minutes at 4°C and the supernatant was transferred to a microfuge tube. Then, 0.2 ml chloroform was added and mixed by inversion for about 30 seconds and incubated at room temperature for 2-3 minutes. Tubes were centrifuged at 12,000×g for 15 minutes at 4°C. The upper layer containing RNA was removed and transferred to a new microfuge tube. In the next stage, RNA precipitation was performed by an equal volume of isopropanol (0.5 ml). For the maximum RNA precipitation, incubation was done at -20°C for 45 minutes (modified protocol). After deposition of RNA, centrifugation was performed at 12,000×g for 20 minutes at 4°C. Supernatant was removed and the pellet was washed with 75% ethanol and centrifuged at 7,500×g for 5 minutes. Finally, ethanol was removed and RNA was dried at room temperature (not completely) and dissolved in an appropriate volume of DEPC treated water. 1% agarose gel electrophoresis was performed to evaluate the quality and integrity of isolated RNA. The RNA concentration and purity was determined using a NanoDrop UV spectrophotometer (BioTek Laboratories, Inc., USA).

*cDNA Synthesis*

RevertAid first strand cDNA synthesis kit (Fermentas, Burlington, ON, Canada) was used for cDNA synthesis. Required mixture was prepared for the reverse transcription reaction in a 20 μl volume as follows: initially, 1 μl template RNA (5 μg) plus 1 μl random hexamer primer (15 pmol) was added into a sterile, nuclease-free tube and reached a 12 μl total volume with DEPC water and incubated at 65°C for 5 minutes. The reaction mixture in total volume of 20 μl was prepared containing: 4 μl 5X Reaction Buffer, 1 μl RiboLock RNase Inhibitor (20 U/μl), 2 μl 10 mM dNTP Mix, and 1 μl RevertAid M-MuLV Reverse Transcriptase (200 U/μl). The prepared mixture incubated in a thermocycler device (Eppendorf Mastercycler, USA) for 5 minutes at 25°C followed by 60 minutes at 42°C. The reaction terminated by heating at 70°C for 5 minutes (according to the manufacturer’s instructions). Agarose gel electrophoresis (1%) was run to confirm the quality and integrity of the synthesized cDNA. The cDNA concentration and purity was determined using a NanoDrop UV spectrophotometer (BioTek Laboratories, Inc., USA).

*Real-Time Polymerase Chain Reaction (qPCR)*

SYBR Premix Ex Taq II (Tli Rnase H Plus), (TaKaRa, China) on CFX96 real-time PCR detection system (Bio-Rad, USA) was applied to amplify and determine mRNA expression levels of SNARE proteins.^17^ 18S rRNA housekeeping gene was used as an internal control.^18^ The forward and reverse primers used for 18S rRNA were: 5`-dGTAACCCGTTGAACCCCATT and 5`-dCCATCCAATCGGTAGTAGCG. Gene-specific primers were designed using AlleleID7 software (Premier Biosoft Corporation, USA). To use in reaction, primers were prepared at a concentration of 10 µM. Primer pairs properties are listed in table 1. qPCR reaction mixture was prepared according to the manufacturer’s instructions for each sample as follows: 10 μl SYBR Premix Ex Taq II (1X), 1 μl of each primer (0.4 μM), 1 μl cDNA template (<100 ng) and 7.0 μl distilled water (20 μl final volume). The reaction was carried out for each sample as triplex and thermal cycling was performed in 40 cycles.

**Table1:** Properties of primer pairs used in real-time RT-PCR

	**Snap23**	**Stx4**	**Vamp2**
NCBI accession number	NM_022689.2	NM_031125.1	NM_012663.2
Forward primer	TTCCGTTTCTGTGTCCAATAG	TCAGCAGACTATGTGGAAC	CTACTTGGTCCTAAGAATCC
Reverse primer	TTGTGCTTTCCAGAGACTCAT	CCAAGATGAGAACAGTGACAGA	CAGAAGAGTGAAGAGTAATGG
Optimized annealing temperature	52.6°C	50.5°C	47.0°C

Snap23: Synaptosomal-associated protein 23; Stx4: Syntaxin 4; Vamp2: Vesicle-associated membrane protein 2

*Insulin Assay*

Rat Insulin (INS) ELISA Kit was used to detect the insulin levels. This kit measured insulin levels in serum samples of rats based on double-antibody sandwich technique (according to supplier’s instruction). Insulin resistance index was calculated as follows: insulin (μU/ml)×glucose (mmol/L)/22.5.^19^

*Statistical Analysis*

The obtained data are presented as mean±SD. In order to statistically compare data between different groups, one way ANOVA followed by post hoc Turkey’s test was used. P values <0.05 were considered significant. The SPSS software (version 16.0) was used for statistical analysis. 

## Results

*The Effect of Resveratrol on Fasting Blood Sugar Levels*

To evaluate and compare the resveratrol effect on FBS levels in different groups, FBS was measured at 3 different times: before induction of diabetes, seven days after diabetes induction, and one month after RSV-treatment. Table 2 shows RSV dose-dependent lowering effect on FBS levels in the RSV-treated groups compared with the health and diabetic groups. Seven days after the induction of diabetes, fasting blood sugar levels were significantly increased in diabetic rats compared with healthy control. Administration of different doses of resveratrol (1, 5, 10 mg/kg) led to decrease FBS in RSV-treated diabetic rats compared with untreated diabetic rats. The results showed that, the lowering effect of resveratrol on FBS was dose dependent. Doses of 5 and 10 mg/kg resveratrol supplementation showed a greater effect on FBS reduction than 1 mg/kg dose. Following the induction of diabetes, insulin levels significantly decreased compared with healthy control. Doses of 5 and 10 mg/kg resveratrol supplementation increased insulin levels to near normal compared to 1 mg/kg dose. Also, as shown in table 2, HOMA index was improved in the groups that received such doses of resveratrol compared with the untreated diabetic group (P=0.01).

**Table 2:** Effect of resveratrol supplementation (RSV) on fasting blood sugar levels and insulin levels

	**Healthy**	**Diabetic**	**Diabetic RSV 1 mg/kg**	**Diabetic RSV 5 mg/kg**	**Diabetic RSV 10 mg/kg**	P value**
Pretreatment day 0	87.3±10.6	95.62±7.3	93.2±14.8	84.1±11.0	90.5±7.23	0.27
7days after diabetes induction	89.12±10.7	279.2±95.3^a#^	289.7±79.2^a#^	219.3±120^a*^	241.2±99.8^a*^	0.001
One month after treatment	92.0±10.8	303.3±92.1^a*^	271.3±77.5^a#^	192.5±84.8^b*^	190.3±68.6^b*^	<0.001
Insulin (μU/ml)	11.17±1.06	7.23±1.15^a#^	8.13±0.97^a#^	9.52±1.05^b#^	9.83±0.86^b#,c*^	<0.001
HOMA	2.51±0.37	5.46±2.09^a*^	5.52±1.92^a*^	3.73±2.01^b#^	4.77±1.08^b#,c*^	0.01

Mean±SD FBS, Insulin and HOMA in healthy (n=8) and diabetic control groups (n=8) and RSV-treated diabetic rats (1, 5, 10 mg/kg, n=8). a: Compared with healthy control; b: Compared with diabetic control; c: Compared with diabetic rats treated with resveratrol 1 mg/kg; ^*^P<0.05; ^#^P<0.001; ^**^Comparison between all groups

*The Effect of Resveratrol on Body Weight of Rats*

Table 3 shows the effect of three doses of resveratrol (1, 5, 10 mg/kg) on rats’ body weight at three time periods: pretreatment, seven days after diabetes induction and one month after treatment. Difference in body weight was significant between the healthy control group and STZ-NA induced diabetic rats (P=0.04). In resveratrol treated diabetic group, an increase in body weight was observed during treatment. However, their body weight did not reach to those of the control group. Also, no significant differences were observed in weight gain between the different doses of RSV (table 3).

**Table 3:** Effect of resveratrol supplementation (RSV) on body weight (g)

	**Healthy**	**Diabetic**	**Diabetic RSV 1 mg/kg**	**Diabetic RSV 5 mg/kg**	**Diabetic RSV 10 mg/kg**	P value**
Pretreatment day 0	218.1±17.1	220.0±14.1	231.2±18.0	228.0±15.0	213.6±18.3	0.20
7days after diabetes induction	229.3±16.7	205.7±14.8^a*^	219.38±17.0^a*^	215.8±7.61^a*^	206.3±17.0^a*^	0.04
One month after treatment	260.6±14.7	191.2±14.4^a#^	222.6±17.6^a#,b^^∆^	221.5±14.9^a#,b^^∆^	211.0±18.0^a#,b^^∆^	<0.001

Mean±SD Body weight in healthy (n=8) and diabetic control groups (n=8) and RSV-treated diabetic rats (1, 5, 10 mg/kg, n=8). a: Compared with healthy control; b: Compared with diabetic control; ^*^P<0.05; ^#^P<0.001; ^∆^: P<0.01; ^**^Comparison between all groups

*Expression of SNAP23, Syntaxin-4 and VAMP-2 in Adipose Tissue*

Expression levels of SNAP23, syntaxin-4 and VAMP-2 were assessed by real-time PCR technique. Table 4 shows the mean delta Ct comparisons in the experimental groups. Gene-specific Ct refers to the number of cycles that fluorescent signal crosses the threshold value. Delta Ct=(Ct value of interest gene)-(Ct value of reference gene). Lower mean delta Ct indicates higher gene expression and vice versa. Results indicated that mean delta Ct of SNAP23, syntaxin-4 and VAMP-2 were higher in diabetic rats than healthy rats, such that the expression of genes was significantly reduced in diabetic rats. Administration of various doses of resveratrol led to increase expression of SNARE proteins in RSV-treated diabetic rats compared with untreated diabetic rats (table 4). Resveratrol was able to increase the expression of all three genes of SNAP23, syntaxin-4 and VAMP-2 in adipose tissue. Such that, resveratrol at doses of 5 mg/kg and 10 mg/kg were able to modulate the altered genes expression close to their expressions in healthy control. Furthermore, there was no significant difference between the two doses in increased expression of SNARE proteins. However, the lowest dose of resveratrol (1 mg/kg) showed most effective on increased expression of SNARE proteins, to the extent that the increase in genes expression levels were much higher than the normal levels. In order to analyze the relative changes between a target and calibrator gene, 2^-ΔΔCt^ method was used in each sample to calculate fold change of expression (table 5).

**Table 4:** Effect of resveratrol supplementation (RSV) on expression of SNAP23, Syntaxin-4 and VAMP-2 (∆ct)

	**Healthy**	**Diabetic**	**Diabetic RSV 1 mg/kg**	**Diabetic RSV 5 mg/kg**	**Diabetic RSV 10 mg/kg**	P value**
Syntaxin-4	12.16±1.10^b#^	15.11±1.58^a#^	11.17±1.60^b#^	13.28±0.77^b#,c*^	12.92±1.44^b#^	<0.001
Vamp-2	8.53±1.82^b#^	11.48±2.7^a#^	6.99±1.40^b#^	9.98±1.55^b#^	9.39±1.40^b#^	<0.001
SNAP-23	4.18±1.42^b#^	8.65±3.3^ a#^	3.98±0.83^b#^	4.98±2.22^b#^	4.78±1.46^b#^	<0.001

Mean±SD ∆Ct in health (n=8), diabetic control groups (n=8) and RSV-treated diabetic rats (1, 5, 10 mg/kg, n=8). a: Compared with healthy control; b: Compared with diabetic control; c: Compared with diabetic rats treated with resveratrol 1 mg/kg; ^*^P<0.05; ^#^P<0.001; ^**^Comparison between all groups

**Table 5:** Fold change of expression of SNARE proteins (2^-ΔΔCt^)

**Groups**	**Syntaxin-4**	**Vamp-2**	**SNAP-23**
Diabetic, Health	0.126	0.129	0.04
RSV 1 mg/kg, Diabetic	15.34	22.47	25
RSV 5 mg/kg, Diabetic	3.55	2.82	12.72
RSV 10 mg/kg, Diabetic	4.5	4.25	14.62

ΔΔCt: ΔCt [case-control]. Comparison of fold change expression between Diabetic and Healthy groups; Diabetic and RSV-treated diabetic rats

## Discussion

GLUT-4 transporter is essential for glucose uptake, which is specifically expressed in two insulin-sensitive tissues i.e. adipose tissue and skeletal muscle. Insulin-stimulated vesicle GLUT-4 trafficking is mediated by SNAREs complex between intracellular compartment and adipocytes surface membrane. Among the various isoforms of SNARE proteins, SNAP-23, VAMP-2 and Syntaxin-4 play important roles in GLUT-4 translocation and therefore glucose uptake in adipocytes.^20^ Based on conducted studies, impaired glucose transport by defects in insulin-mediated GLUT-4 translocation leads to insulin resistance. Since adipose tissue is an insulin-sensitive organ, resistance to insulin, resulting impaired vesicle GLUT-4 transfer affected glucose uptake by adipocytes.^21^ We studied the effect of RSV as a natural antioxidant with anti-diabetic properties on the modified SNARE proteins expression. In this study, animal type 2 diabetes model was induced by STZ-NA injection, which is close to human type 2 diabetes.^22^ Results of various studies have shown that resveratrol can affect diabetes by several mechanisms. However, in different studies, there are contradictions depending on the experimental model, the dose and mode of RSV administration, the type of tissue examined and existing conditions.^11^ Among the effects of RSV that have been demonstrated in diabetes, it can be pointed to its performance in preventing weight loss, reduction of diabetes-specific symptoms and blood glucose levels.^23^ Therefore, consistent with these findings, the results of the current study showed that oral administration of RSV increased body weight and decreased hyperglycemia in diabetic rats treated with RSV compared with untreated diabetic controls. In this study, HOMA index was examined for assessing insulin resistance and the results showed that RSV improved insulin resistance in diabetic rats compared with untreated diabetic controls. There is evidence that RSV enhances insulin secretion and insulin tissue sensitivity in STZ-NA induced diabetic rats. It is proven that, RSV binding to the sulfonylurea receptor in pancreatic β cell membrane is the mechanism of its action on insulin secretion. So that stimulation and activation of these receptors is associated with ATP-sensitive K^+^ channels blockage and therefore plasma membrane depolarization and the subsequent insulin release from pancreatic β cells.^24^^,^^25^ Also, it is shown that RSV antioxidant property decreased the oxidative-stress related damage in diabetic tissues. Oxidative stress caused by STZ destroys pancreatic beta cells, although nicotinamide exert antioxidant effects and led to partial protection of beta cells against the damage caused by STZ and prevent the complete destruction of the insulin-secreting cells. It is proven that, RSV antioxidant property decreased the oxidative-stress related damage in diabetic secretion, which is another mechanism of resveratrol on the insulin secretion by reducing oxidative stress.^26^ It is well known that, the main mechanism of resveratrol on glucose homeostasis and insulin sensitivity is via the activation of sirtuin 1 protein (SIRT1), which is an activator for GLUT-4 transporter mediated AMPK activation.^27^ Some studies have pointed to resveratrol’s role on GLUT-4 increased expression, activation and translocation in diabetic animals.^11^ According to SNARE proteins involved in vesicle GLUT-4 trafficking in insulin-sensitive tissues, our study examined the effect of resveratrol on altered expression of this proteins in adipose tissue of diabetic rats. In a study that evaluated the rosiglitazone effect on SNAREs proteins expression in hyperinsulinemia and insulin resistance status in the ZDF rat model, increased expression of these proteins was observed, while did not find significant differences in STZ-induced diabetic rats under conditions of hyperglycemia in the absence of insulin.^9^ Another study reported a decrease in transport and GLUT-4 content in adipose tissue of diabetic rats induced by STZ.^28^

Ostenson et al., studying in pancreatic islets of type 2 diabetic patients, showed impairment of SNARE proteins gene expression and concluded that these changes lead to diminish insulin secretion.^29^

Our results, consistent with previous studies, showed significant changes in the SNARE proteins expression in diabetic rats compared to controls. However, unlike these studies, the results of our study demonstrated a significant reduction in the SNARE proteins expression in diabetic rats compared to controls. As the expression of genes related to SNAP-23, VAMP-2 and Syntaxin-4 dramatically reduced in adipose tissue of diabetic rats compared with normal rats. The difference between the results of the present study with previously mentioned studies is probably due to the partial insulin release from pancreatic β cells and moderate hyperglycemia in STZ-NA induced diabetic rats. Therefore, the glucose tolerance is created in the same conditions might be due to the reduced expression of the involved genes in GLUT-4 transport and impaired membrane GLUT-4 transfer in adipocytes. 

Interestingly, our study demonstrated a dose-dependent modulatory effect of RSV on the expression of SNAP-23, VAMP-2 and Syntaxin-4 in diabetic rats treated with resveratrol during a period of 30 days. As RSV at doses of 5 and 10 mg/kg were able to return the reduced expression of these genes to normal or near-normal levels. Unexpected point of this study was that the lowest dose of RSV (1 mg/kg) was more effective in increasing the expression of SNARE proteins compared with other doses. It is likely that 1 mg/kg resveratrol dosage was not able to apply its mechanisms of action in the current situation and therefore the increased expression more than normal occurs as a compensatory mechanism. The effect of RSV in expression of other genes also has been studied. Rahvar et al. showed that RSV increases BDNF gene expression and concluded that this effect can have a neuroprotective role.^30^

## Conclusion

The results of this study indicate that STZ-NA induced diabetes associated with decreased SNARE proteins expression that could disrupt GLUT-4 transport and insulin resistance. According to the findings of this study, another mechanism of resveratrol effect could be suggested as reducing blood glucose by adjusting the altered expression of these genes.  
